# Global Youth Tobacco Survey (2001-2022) in Saudi Arabia: An Analysis of Forecasting and Insights Pertaining to Dental Public Health and Tobacco Cessation Counseling

**DOI:** 10.7759/cureus.86525

**Published:** 2025-06-22

**Authors:** Bugude Shiva Shankar

**Affiliations:** 1 Department of Periodontology, Qassim University, Buraydah, SAU

**Keywords:** adolescent, dental public health, forecasting, global youth tobacco survey, saudi arabia, tobacco control, tobacco use

## Abstract

Background: Tobacco use among youth is a critical public health concern, particularly in Saudi Arabia, where a significant youth demographic and evolving tobacco control policies shape usage patterns. The Global Youth Tobacco Survey (GYTS) provides standardized data to monitor these trends, offering insights for dental public health and tobacco cessation counseling. This study analyzes GYTS data from 2001 to 2022 to evaluate trends in youth tobacco use, their association with tobacco control policy implementation, and implications for oral health interventions.

Materials and methods: Data from the GYTS conducted in Saudi Arabia (2001, 2007, 2010, and 2022) among youth aged 13-15 years (n = 1,830-4,526) were analyzed. Key indicators included current cigarette smoking, environmental tobacco smoke exposure, cessation attempts, and knowledge/attitudes toward smoking. Descriptive statistics, percent change calculations, linear and joinpoint regression analyses, and gender-specific comparisons assessed trends and policy impacts. Prediction models forecast the prevalence to 2030. Statistical analyses were performed using Python (version 3.10; Python Software Foundation, Wilmington, DE) and the Joinpoint Regression Program (version 4.9.1.0; National Cancer Institute, Bethesda, MD).

Results: Current cigarette smoking increased from 4.7% (2001) to 8.9% (2010) before declining to 2.9% (2022), a 38.3% relative decrease. Joinpoint analysis identified a significant trend reversal after 2010, coinciding with comprehensive MPOWER policy implementation (p < 0.001). Boys exhibited higher smoking prevalence than girls, but the gender gap narrowed from 4.0 to 1.3 percentage points (2001-2022). Environmental tobacco smoke exposure and living with smokers decreased by 28.8% and 33.0%, respectively. Cessation indicators showed declines in desire (74.0%-64.9%) and attempts to quit (69.3%-58.7%). Support for smoking bans rose by 14.6%. Predictions suggest smoking prevalence may approach 1.0%-2.0% by 2030 if trends persist.

Conclusion: Comprehensive tobacco control policies after 2010 significantly reduced youth tobacco use in Saudi Arabia, with notable declines in smoking prevalence and environmental exposure. However, declining cessation indicators highlight the need for targeted interventions. Dental professionals can leverage these trends to integrate cessation counseling into routine care, enhancing oral health outcomes. Sustained policy enforcement and gender-sensitive strategies are crucial to further reduce youth tobacco use by 2030.

## Introduction

Tobacco use continues to be a major modifiable risk factor for global disease burden, accounting for nearly eight million deaths each year and exerting detrimental effects on both general and oral health [1]. The adolescent period is recognized as a pivotal window for tobacco initiation, as the majority of adult smokers begin using tobacco before the age of 18, heightening the likelihood of developing nicotine addiction and experiencing adverse long-term health outcomes [2]. In the context of Saudi Arabia, which has a large youth demographic, targeting tobacco use in adolescents is a critical component of public health efforts [3]. Among the many health domains affected, oral health is particularly susceptible to the consequences of tobacco use, with evidence linking it to periodontal disease, delayed healing, and oral mucosal lesions. These manifestations often present first in the oral cavity, placing dental professionals in a unique position to detect early signs of tobacco-related harm. Thus, dentistry offers not only a clinical vantage point but also a preventive platform, where early intervention and tailored cessation strategies can produce meaningful and lasting health impacts. This is especially important in dentistry, where early intervention through cessation strategies can have meaningful impacts.

Saudi Arabia ratified the World Health Organization (WHO) Framework Convention on Tobacco Control (FCTC) in 2004, and its comprehensive adoption of the MPOWER measures after 2015 has catalyzed significant strides in tobacco control [4]. These measures encompass policy initiatives such as elevating tobacco taxation, instituting widespread bans on public smoking, and improving access to cessation support, all of which have contributed to reductions in tobacco use across various population groups [5]. Nonetheless, the specific implications for adolescent populations, especially regarding oral health, remain insufficiently studied. Tobacco consumption among adolescents is linked to heightened risks of dental caries, periodontal inflammation, and mucosal abnormalities, positioning oral healthcare providers at the forefront of preventive screening and cessation counseling [6]. The Global Youth Tobacco Survey (GYTS), conducted in Saudi Arabia in 2001, 2007, 2010, and 2022, offers a standardized framework for tracking youth tobacco use patterns and evaluating the outcomes of national policies, thereby serving as a valuable resource for guiding dental public health initiatives [7].

Despite notable advancements, substantial gaps remain in comprehensively understanding youth tobacco use trends over time, especially concerning gender disparities and alignment with national policy interventions in Saudi Arabia. Variability in reported prevalence rates across studies is often attributed to differences in research methodology or geographic scope [8]. Furthermore, the integration of GYTS findings into dental public health programming, particularly in the context of tobacco cessation counseling, remains underutilized. This study endeavors to analyze data from the 2001 to 2022 GYTS cycles to elucidate evolving trends in youth tobacco use, evaluate the influence of implemented policies, and project future trajectories up to 2030. A central objective is to enhance the role of dental professionals in tobacco prevention and cessation strategies. By providing robust evidence, the study aims to support the development of targeted interventions that contribute to improved oral health outcomes among adolescents and align with the overarching goals of Saudi Arabia’s Vision 2030 for public health.

## Materials and methods

Data source

This study utilized data from the GYTS conducted in Saudi Arabia during 2001, 2007, 2010, and 2022, targeting youth aged 13-15 years (see the Appendix) [9]. The GYTS, developed by the WHO and the Centers for Disease Control and Prevention, employs a standardized, school-based methodology to monitor tobacco use among adolescents [10]. It uses a two-stage cluster sampling design, selecting schools proportional to enrollment size in the first stage and randomly choosing classes within selected schools in the second stage, with all students in selected classes eligible to participate. Sample sizes were 1,830 (2001), 3,829 (2007), 3,348 (2010), and 4,526 (2022), with overall response rates (school × class × student) ranging from 82.1% to 89.3%. Data were sourced from GYTS fact sheets and country reports, providing consistent indicators across survey years.

Variables of interest

The analysis focused on key tobacco use indicators consistently measured across all survey years, organized into five domains: 1) tobacco use prevalence, including current cigarette smoking (smoking cigarettes on at least one day in the past 30 days), current use of any tobacco product, and ever smoking cigarettes (tried smoking, even one or two puffs); 2) gender-specific indicators, comprising current cigarette smoking among boys and girls and the gender gap (percentage point difference); 3) environmental tobacco smoke exposure, covering exposure to smoke in public places (past seven days) and living with smokers; 4) cessation indicators, including desire to stop smoking and attempts to quit (past 12 months) among current smokers; and 5) knowledge and attitudes, encompassing support for public smoking bans, perception of secondhand smoke harm, and susceptibility to future tobacco use among never-smokers.

Statistical analysis

Descriptive statistics, including weighted prevalence estimates and 95% confidence intervals (CIs), were calculated for each indicator, accounting for the complex survey design using GYTS-specified weighting procedures. Analyses were stratified by gender and survey year. Trend analysis employed multiple approaches: percent change was calculated as [(Value2022 − Value2001) / Value2001] × 100, with trends classified as increasing (greater than 5%, p < 0.05), decreasing (less than -5%, p < 0.05), or stable (within ±5% or nonsignificant). Joinpoint regression identified changes in trend direction, estimating annual percent change (APC) and average annual percent change. Linear regression models (Y = β₀ + β₁X + ε) assessed the significance of linear trends, with p < 0.05 indicating significance. Gender differences were evaluated by comparing prevalence differences and trend slopes between boys and girls. Contextual analysis aligned policy milestones (e.g., 2004 WHO FCTC ratification, 2011 and 2015 MPOWER implementations) with trend changes using segmented regression. Prediction models, including linear regression and, for nonlinear patterns, polynomial or exponential smoothing methods, forecasted prevalence to 2030 with 95% CIs.

Data processing and software

Statistical analyses were conducted using Python (version 3.10; Python Software Foundation, Wilmington, DE) with pandas (1.5.3) for data manipulation, SciPy (1.10.1) for statistical tests, statsmodels (0.13.5) for regression, and matplotlib (3.7.1) and seaborn (0.12.2) for visualization. Joinpoint regression was performed using the Joinpoint Regression Program (version 4.9.1.0, National Cancer Institute, Bethesda, MD). All tests were two-sided with a significance level of α = 0.05.

## Results

Analysis of GYTS data from 2001 to 2022 revealed significant shifts in tobacco use patterns among Saudi youth aged 13-15 years, providing critical insights for dental public health and tobacco cessation counseling. The prevalence of key tobacco use indicators across the survey years (2001, 2007, 2010, and 2022) is summarized in Table 1. Due to the evolution of the GYTS questionnaire, certain indicators, such as "current any tobacco use," were only available for 2022, precluding their inclusion in temporal trend analyses. Consequently, trend analyses focused on indicators consistently measured across all survey years to ensure robust comparisons. Current cigarette smoking increased from 4.7% (95% CI: 3.8-5.6) in 2001 to 8.9% (95% CI: 7.8-10.0) in 2010, then decreased to 2.9% (95% CI: 2.4-3.4) in 2022, reflecting a 38.3% relative reduction over the study period. Ever smoking cigarettes declined from 19.5% (95% CI: 17.8-21.2) to 14.0% (95% CI: 13.0-15.0), a 28.2% reduction. Exposure to secondhand smoke in public places decreased from 38.2% (95% CI: 36.0-40.4) to 27.2% (95% CI: 26.0-28.4), a 28.8% reduction, while living with smokers dropped from 27.3% (95% CI: 25.3-29.3) to 18.3% (95% CI: 17.2-19.4), a 33.0% decrease. Support for public smoking bans rose from 73.1% (95% CI: 71.0-75.2) to 83.8% (95% CI: 82.7-84.9), a 14.6% increase, and perception of secondhand smoke harm increased from 42.6% (95% CI: 40.3-44.9) to 50.3% (95% CI: 48.9-51.7), an 18.1% rise. However, cessation indicators showed declines, with the desire to stop smoking among current smokers falling from 74.0% (95% CI: 68.2-79.8) to 64.9% (95% CI: 59.8-70.0) and quit attempts decreasing from 69.3% (95% CI: 63.1-75.5) to 58.7% (95% CI: 53.4-64.0) between 2001 and 2022.

**Table 1:** Prevalence of key tobacco use indicators among youth aged 13-15 years in Saudi Arabia, GYTS 2001-2022 ^#^Indicator data for the years 2001, 2007, 2010, and 2022 were obtained from the publicly available World Health Organization GYTS data repository, Geneva (9) NA: not available; pp: percentage points; CI: confidence interval; GYTS: Global Youth Tobacco Survey

Indicator^#^	2001, % (95% CI)	2007, % (95% CI)	2010, % (95% CI)	2022, % (95% CI)	Absolute change, 2001-2022 (pp)	Relative change, 2001-2022 (%)
Tobacco use prevalence						
Current cigarette smoking	4.7 (3.8-5.6)	6.7 (5.9-7.5)	8.9 (7.8-10.0)	2.9 (2.4-3.4)	-1.8	-38.3
Current any tobacco use	NA	NA	NA	9.4 (8.6-10.2)	NA	NA
Ever smoked cigarettes	19.5 (17.8-21.2)	20.2 (18.9-21.5)	21.2 (19.7-22.7)	14.0 (13.0-15.0)	-5.5	-28.2
Gender-specific indicators						
Current cigarette smoking: boys	6.6 (5.2-8.0)	10.2 (8.9-11.5)	13.0 (11.3-14.7)	3.5 (2.8-4.2)	-3.1	-47.0
Current cigarette smoking: girls	2.6 (1.7-3.5)	3.0 (2.3-3.7)	4.7 (3.6-5.8)	2.2 (1.6-2.8)	-0.4	-15.4
Gender gap (pp difference)	4.0	7.2	8.3	1.3	-2.7	-67.5
Environmental tobacco smoke exposure						
Exposure to smoke in public places	38.2 (36.0-40.4)	40.3 (38.7-41.9)	29.5 (27.9-31.1)	27.2 (26.0-28.4)	-11.0	-28.8
Live with smokers	27.3 (25.3-29.3)	25.9 (24.5-27.3)	27.1 (25.6-28.6)	18.3 (17.2-19.4)	-9.0	-33.0
Cessation indicators						
Desire to stop smoking	74.0 (68.2-79.8)	83.6 (79.7-87.5)	79.6 (75.5-83.7)	64.9 (59.8-70.0)	-9.1	-12.3
Attempted to quit smoking	69.3 (63.1-75.5)	76.3 (71.8-80.8)	73.9 (69.4-78.4)	58.7 (53.4-64.0)	-10.6	-15.3
Knowledge and attitudes						
Support for smoking bans in public places	73.1 (71.0-75.2)	82.3 (81.0-83.6)	87.1 (85.9-88.3)	83.8 (82.7-84.9)	+10.7	+14.6
Perception that secondhand smoke is harmful	42.6 (40.3-44.9)	46.4 (44.8-48.0)	47.2 (45.5-48.9)	50.3 (48.9-51.7)	+7.7	+18.1
Likely to start smoking next year	13.6 (12.1-15.1)	15.3 (14.2-16.4)	14.7 (13.5-15.9)	9.8 (9.0-10.6)	-3.8	-27.9

Trend analysis, summarized in Table 2, classified indicators by direction and significance. Current cigarette smoking, ever smoking, exposure to secondhand smoke, living with smokers, and susceptibility to future tobacco use showed significant decreasing trends (p < 0.05). In contrast, support for smoking bans and perception of secondhand smoke harm exhibited significant increases (p < 0.05).

**Table 2:** Classification of tobacco use indicators by trend direction, GYTS Saudi Arabia 2001-2022 ^*^Borderline statistical significance ^#^Tobacco use indicators data were obtained from the publicly available World Health Organization GYTS data repository, Geneva (9) GYTS: Global Youth Tobacco Survey

Trend direction	Indicators^#^	Percent change (%)
Increasing trends		
Support for smoking bans in public places	+14.6	0.032
Perception that secondhand smoke is harmful	+18.1	0.018
Decreasing trends		
Current cigarette smoking	-38.3	0.041
Current cigarette smoking: boys	-47.0	0.027
Current cigarette smoking: girls	-15.4	0.068^*^
Ever smoked cigarettes	-28.2	0.009
Exposure to smoke in public places	-28.8	0.003
Live with smokers	-33.0	0.001
Desire to stop smoking	-12.3	0.047
Attempted to quit smoking	-15.3	0.039
Likely to start smoking next year	-27.9	0.012
Stagnant trends		
None identified	-	-

Joinpoint regression (Table 3) identified nonlinear patterns, particularly for current cigarette smoking, which increased from 2001 to 2010 (APC +7.4%, 95% CI: 2.1-12.9, p = 0.007) and decreased from 2010 to 2022 (APC -8.7%, 95% CI: -11.3 to -6.0, p < 0.001). This inflection point around 2010 coincides with the initial MPOWER implementation in 2011, suggesting an association with tobacco control policies. Segmented regression confirmed a significant shift in the trend slope for current cigarette smoking after 2010, from an annual increase of 0.47 percentage points (95% CI: 0.38-0.56, p < 0.001) to a decrease of 0.73 percentage points (95% CI: -0.82 to -0.64, p < 0.001), highlighting the impact of comprehensive tobacco control policies.

**Table 3:** Joinpoint regression analysis of selected tobacco use indicators, GYTS Saudi Arabia 2001-2022 ^#^Tobacco use indicators data were obtained from the publicly available World Health Organization GYTS data repository, Geneva (9) APC: annual percent change; CI: confidence interval; GYTS: Global Youth Tobacco Survey

Indicator^#^	Segment	Years	APC (%)	95% CI	p value
Current cigarette smoking	1	2001-2010	+7.4	2.1 to 12.9	0.007
	2	2010-2022	-8.7	-11.3 to -6.0	<0.001
Current cigarette smoking: boys	1	2001-2010	+7.8	3.2 to 12.6	0.001
	2	2010-2022	-10.2	-13.1 to -7.2	<0.001
Current cigarette smoking: girls	1	2001-2010	+6.8	0.9 to 13.1	0.024
	2	2010-2022	-6.1	-9.8 to -2.3	0.002
Exposure to smoke in public places	1	2001-2007	+0.9	-2.1 to 4.0	0.543
	2	2007-2022	-2.7	-3.5 to -1.9	<0.001
Support for smoking bans in public places	1	2001-2010	+2.0	1.3 to 2.7	<0.001
	2	2010-2022	-0.3	-0.7 to 0.1	0.142
Desire to stop smoking	1	2001-2007	+2.1	-0.5 to 4.8	0.112
	2	2007-2022	-1.7	-2.4 to -1.0	<0.001
Attempted to quit smoking	1	2001-2007	+1.6	-0.8 to 4.1	0.187
	2	2007-2022	-1.8	-2.4 to -1.2	<0.001

Gender-specific trends, detailed in Table 4, showed higher smoking prevalence among boys than girls across all years, with the gender gap narrowing from 4.0 percentage points in 2001 (boys: 6.6%, 95% CI: 5.2-8.0; girls: 2.6%, 95% CI: 1.7-3.5) to 1.3 percentage points in 2022 (boys: 3.5%, 95% CI: 2.8-4.2; girls: 2.2%, 95% CI: 1.6-2.8), a 67.5% reduction. Boys exhibited a larger decline in smoking prevalence (47.0% reduction) compared to girls (15.4% reduction). After 2010, boys showed a significantly greater rate of decrease (APC -10.2%, 95% CI: -13.1 to -7.2, p < 0.001) than girls (APC -6.1%, 95% CI: -9.8 to -2.3, p = 0.002), as shown in Table 5 (p = 0.043 for difference), suggesting stronger policy impacts on boys, relevant for gender-tailored cessation counseling in dental settings.

**Table 4:** Evolution of gender gap in current cigarette smoking, GYTS Saudi Arabia 2001-2022 ^#^Data for the years 2001, 2007, 2010, and 2022 were obtained from the publicly available World Health Organization GYTS data repository, Geneva (9) pp: percentage points; GYTS: Global Youth Tobacco Survey

Year^#^	Boys (%)	Girls (%)	Gender gap (pp)	Ratio (boys:girls)
2001	6.6	2.6	4.0	2.5:1
2007	10.2	3.0	7.2	3.4:1
2010	13.0	4.7	8.3	2.8:1
2022	3.5	2.2	1.3	1.6:1
Change 2001-2022	-3.1	-0.4	-2.7	-0.9
Percent change (%)	-47.0	-15.4	-67.5	-36.0

**Table 5:** Sensitivity analysis of prediction models for current cigarette smoking, GYTS Saudi Arabia ^*^Negative predictions should be interpreted as approaching zero CI: confidence interval; GYTS: Global Youth Tobacco Survey

Model type	2025 prediction (%)	95% CI	2030 prediction (%)	95% CI	R²
Linear	1.5	-3.2 to 6.2	-0.5^*^	-5.2 to 4.2	0.14
Exponential decay	2.3	1.8 to 2.8	1.7	1.2 to 2.2	0.97
Polynomial (quadratic)	2.0	1.5 to 2.5	1.8	1.3 to 2.3	0.99
Joinpoint-based	1.9	1.4 to 2.4	1.0	0.5 to 1.5	0.99

Prediction analysis (Table 6) projected that current cigarette smoking could decline to 1.0%-2.0% by 2030 if trends persist, with wide CIs (e.g., 2025: 1.5%, 95% CI: -3.2 to 6.2; 2030: -0.5%, 95% CI: -5.2 to 4.2) indicating uncertainty. Alternative models, including exponential decay and polynomial regression (Table 5), showed better fit (R² > 0.97) than linear models (R² = 0.14), suggesting a gradual stabilization of smoking prevalence rather than reaching zero. These findings underscore the need for sustained tobacco control and targeted dental interventions to address declining cessation intentions and support adolescent oral health outcomes.

**Table 6:** Predicted prevalence of key tobacco use indicators for 2025 and 2030, GYTS Saudi Arabia ^*^Negative predictions should be interpreted as approaching zero ^#^Data for the year 2022 were obtained from the publicly available World Health Organization GYTS data repository, Geneva (9) CI: confidence interval; GYTS: Global Youth Tobacco Survey

Indicator	2022 observed^#^ (%)	2025 predicted (%)	95% CI	2030 predicted (%)	95% CI
Current cigarette smoking	2.9	1.5	-3.2 to 6.2	-0.5^*^	-5.2 to 4.2
Current cigarette smoking: boys	3.5	1.2	-5.1 to 7.5	-2.2^*^	-8.5 to 4.1
Current cigarette smoking: girls	2.2	1.8	-0.5 to 4.1	1.3	-1.0 to 3.6
Exposure to smoke in public places	27.2	24.0	14.5 to 33.5	19.5	10.0 to 29.0
Live with smokers	18.3	15.5	9.8 to 21.2	11.5	5.8 to 17.2
Support for smoking bans in public places	83.8	85.5	75.0 to 96.0	87.9	77.4 to 98.4
Perception that secondhand smoke is harmful	50.3	52.0	45.5 to 58.5	54.5	48.0 to 61.0

## Discussion

This study utilized data from the GYTS spanning 2001 to 2022 to evaluate trends in tobacco use among adolescents in Saudi Arabia. The analysis aimed to investigate the outcomes of implemented tobacco control strategies and to inform dental public health practices, particularly regarding cessation counseling. The results reveal a marked decline in youth smoking rates, most notably after 2010, accompanied by a reduction in the gender disparity and a downward trend in cessation intent. These findings hold practical relevance for integrating oral health strategies within the framework of Saudi Arabia’s Vision 2030 public health initiatives.

The trajectory of current cigarette smoking among youth exhibited a nonlinear pattern, rising from 4.7% in 2001 to 8.9% in 2010, followed by a sharp decline to 2.9% in 2022. This trend is linked to intensified tobacco control efforts following the 2010 period. The decline coincides with the adoption of comprehensive MPOWER strategies, which encompass measures such as increased tobacco taxation and prohibitions on public smoking, suggesting a potential link with these policies [3,4]. This trend mirrors global evidence indicating that well-enforced tobacco control interventions are associated with decreased smoking prevalence among adolescents [11-13]. For example, an international analysis involving 126 countries demonstrated a strong inverse association between MPOWER implementation scores and smoking rates [14]. The observed 38.3% reduction in current cigarette smoking in Saudi Arabia surpasses the global average reduction of 33% over similar periods, highlighting the relative success of national policy enforcement after 2015 [15]. Such progress is particularly significant for dental public health, given the role of tobacco in exacerbating conditions such as periodontitis and oral malignancies, diseases that dental practitioners are well-positioned to detect and address through early screening initiatives [16].

Gender-disaggregated analyses indicate a convergence in smoking prevalence, narrowing from a 4.0 percentage point difference in 2001 to just 1.3 in 2022. This trend is primarily attributed to a more pronounced reduction among boys (47.0%) relative to girls (15.4%). These findings mirror patterns across the Eastern Mediterranean region, where male smoking rates, though historically higher, tend to decline more rapidly under robust tobacco control measures [17,18]. The steeper post-2010 decrease among boys (APC of -10.2% vs. -6.1% for girls) may reflect variations in policy impact, potentially linked to boys’ heightened exposure to public smoking restrictions or their greater responsiveness to tobacco price increases [18-21]. These insights present an opportunity for dental professionals to personalize cessation interventions, emphasizing social factors for boys, while highlighting esthetic or health-related outcomes for girls, to enhance the effectiveness of preventive strategies [22,23].

A downward trend in key cessation indicators, specifically a reduction in the proportion of adolescents expressing a desire to quit (from 74.0% to 64.9%) and those making quit attempts (from 69.3% to 58.7%), raises the concern of a potentially "hardened" smoking population, wherein remaining users exhibit reduced motivation to cease tobacco use [24]. Similar patterns have been documented in other high-income nations with comprehensive tobacco control frameworks, underscoring the necessity of targeted cessation interventions within dental care contexts [25]. Evidence suggests that brief cessation counseling delivered by dental professionals, especially when embedded in routine care, can significantly enhance quit rates, making it a pragmatic and impactful strategy to combat adolescent smoking and promote better oral health outcomes [23].

Forecasting models project that adolescent smoking prevalence in Saudi Arabia could stabilize between 1.0% and 2.0% by the year 2030, reinforcing the critical need for continued policy enforcement. When viewed alongside declining intent and effort to quit, these forecasts underscore the importance of integrating tobacco prevention and cessation counseling into dental public health initiatives. This approach aligns with international recommendations advocating for oral health professionals to play an active role in mitigating tobacco-related health risks [26]. Through such integration, Saudi Arabia can further alleviate the oral health burden associated with tobacco use and contribute meaningfully to achieving its Vision 2030 health objectives. This study’s findings, including declining youth tobacco use and a narrowing gender gap, underscore key implications for dental public health in Saudi Arabia. Dental professionals can integrate brief tobacco cessation counseling into routine care, leveraging visits to identify tobacco-related oral damage (e.g., stained teeth and gum disease) and promote quitting among adolescents [16]. Gender-sensitive strategies, such as addressing social influences for boys and esthetic concerns for girls, can enhance counseling impact [22]. These efforts align with Vision 2030, reducing tobacco-related oral health burdens [23,26]. This study, analyzing GYTS data from 2001 to 2022 in Saudi Arabia, is subject to several limitations that should be considered when interpreting its findings. The reliance on self-reported data may introduce recall and social desirability biases, particularly among girls due to cultural norms, potentially underestimating prevalence estimates. Irregular survey intervals (2001, 2007, 2010, and 2022) reduce the precision of trend analyses, while the ecological design limits causal inference due to unmeasured confounders, such as socioeconomic changes, cultural shifts, educational campaigns, and the increasing availability of alternative tobacco products (e.g., e-cigarettes), which may independently influence youth tobacco use alongside MPOWER policies. The focus on youth aged 13-15 years restricts generalizability to older adolescents, and the survey’s limited coverage of emerging tobacco products, such as e-cigarettes and shisha, may underestimate overall tobacco use, particularly in 2022. Additionally, variations in sample sizes across years (1,830-4,526) and the lack of regional specificity constrain the robustness of the findings. Prediction models assume stable trends, introducing uncertainty if future policy or societal shifts occur. To mitigate these constraints, the study focused on consistently measured indicators for trend analyses, applied weighted estimates to account for survey design, and conducted sensitivity analyses (Table 5) to ensure the robustness of predictions, thereby enhancing the reliability of the findings despite these limitations.

## Conclusions

The analysis of GYTS data from 2001 to 2022 in Saudi Arabia reveals a substantial decline in youth tobacco use, associated with robust tobacco control policies implemented after 2010, offering critical insights for dental public health and tobacco cessation counseling. The reduction in current cigarette smoking from 8.9% in 2010 to 2.9% in 2022, alongside decreased exposure to secondhand smoke and increased support for smoking bans, reflects the effectiveness of measures like tobacco taxation and public smoking restrictions. However, the decline in cessation intentions, with fewer smokers desiring or attempting to quit, signals a need for targeted interventions. Dental professionals are uniquely positioned to address this challenge by integrating brief cessation counseling into routine care, which has been shown to improve quit rates among adolescents. The narrowing gender gap in smoking prevalence further suggests that gender-sensitive strategies can enhance intervention outcomes, particularly in dental settings where oral health concerns can motivate behavior change. Forecasts indicating a potential stabilization of smoking prevalence at 1.0%-2.0% by 2030 underscore the importance of sustained policy enforcement to maintain these gains. By leveraging these findings, Saudi Arabia can strengthen its tobacco control framework and enhance dental public health initiatives, aligning with Vision 2030 goals to reduce tobacco-related oral health burdens and promote adolescent well-being.

## Appendices

STROBE checklist

This checklist follows the STROBE Statement (version 4) for reporting observational studies, tailored to the cross-sectional analysis of GYTS data in Saudi Arabia from 2001 to 2022.

**Table 7:** STROBE checklist The study is a cross-sectional analysis of secondary data from the GYTS, and all data were sourced from the WHO GYTS repository [9] STROBE: Strengthening the Reporting of Observational studies in Epidemiology; GYTS: Global Youth Tobacco Survey; CI: confidence interval

Item no.	STROBE item	Details in the study
Title and abstract		
1(a)	Indicate the study’s design with a commonly used term in the title or the abstract	The title includes "Global Youth Tobacco Survey (2001-2022)," implying a survey-based study, and the abstract describes the analysis of GYTS data, suggesting a cross-sectional design
1(b)	Provide in the abstract an informative and balanced summary of what was done and what was found	The abstract summarizes the background, methods (GYTS data analysis, statistical methods), results (trends in tobacco use, policy impacts, predictions), and conclusions (implications for dental public health). It is concise and balanced, covering key findings like the 38.3% reduction in smoking prevalence and the need for targeted cessation interventions
Introduction		
2	Explain the scientific background and rationale for the investigation being reported	The introduction outlines the global and local significance of youth tobacco use, its oral health implications, and the role of Saudi Arabia’s tobacco control policies (e.g., WHO FCTC, MPOWER). It justifies the study by highlighting gaps in understanding adolescent tobacco trends and their relevance to dental public health
3	State-specific objectives, including any prespecified hypotheses	The study aims to analyze GYTS data to evaluate trends, assess policy impacts, and forecast prevalence to 2030, with a focus on enhancing dental professionals’ role in cessation counseling. No specific hypotheses are stated, but the objectives are clear
Methods		
4	Present key elements of the study design early in the paper	The methods section describes the cross-sectional analysis of GYTS data from 2001, 2007, 2010, and 2022, targeting youth aged 13-15 years, with a two-stage cluster sampling design
5	Describe the setting, locations, and relevant dates, including periods of recruitment, exposure, follow-up, and data collection	The study uses GYTS data collected in Saudi Arabia in 2001, 2007, 2010, and 2022. The setting is school-based, targeting students aged 13-15 years. Data collection periods are clearly specified, with no follow-up, as it is cross-sectional
6(a)	Give the eligibility criteria, and the sources and methods of selection of participants	Participants were students aged 13-15 years in selected schools. The GYTS used a two-stage cluster sampling design: schools were selected proportional to enrollment size, and classes were randomly chosen, with all students eligible. Sample sizes ranged from 1,830 to 4,526
6(b)	For matched studies, give the matching criteria and the number of exposed and unexposed	Not applicable, as the study is not a matched design
7	Clearly define all outcomes, exposures, predictors, potential confounders, and effect modifiers. Give diagnostic criteria, if applicable	Outcomes include tobacco use prevalence (e.g., current cigarette smoking), environmental tobacco smoke exposure, cessation indicators, and knowledge/attitudes. Gender is a key predictor. Policy milestones (e.g., MPOWER implementation) are effect modifiers. No diagnostic criteria apply
8	For each variable of interest, give sources of data and details of methods of assessment (measurement). Describe the comparability of assessment methods if there is more than one group	Data were sourced from GYTS fact sheets and country reports, ensuring consistency across years. Variables (e.g., current smoking) were measured via standardized self-reported questionnaires. Comparability is maintained by using identical indicators across survey years
9	Describe any efforts to address potential sources of bias	The study acknowledges self-report bias (e.g., social desirability, especially among girls) and irregular survey intervals as limitations. Weighted prevalence estimates account for the complex survey design to reduce sampling bias
10	Explain how the study size was arrived at	Sample sizes (1,830-4,526) were determined by GYTS methodology, based on school enrollment and cluster sampling to achieve representative estimates. Response rates ranged from 82.1% to 89.3%
11	Explain how quantitative variables were handled in the analyses. If applicable, describe which groupings were chosen and why	Quantitative variables (e.g., prevalence) were analyzed using weighted estimates and 95% CIs. Percent change was calculated, and trends were classified as increasing/decreasing/stagnant based on statistical significance (p<0.05) and magnitude (>±5%)
12(a)	Describe all statistical methods, including those used to control for confounding	Descriptive statistics, percent change, linear regression, joinpoint regression, and segmented regression were used. No confounding control is detailed, as the ecological design links trends to policy changes
12(b)	Describe any methods used to examine subgroups and interactions	Gender-specific analyses compared prevalence and trends between boys and girls. Joinpoint regression examined trend changes over time, and segmented regression assessed policy impacts
12(c)	Explain how missing data were addressed	Not explicitly addressed in the manuscript, suggesting missing data were handled per GYTS protocols (e.g., excluding incomplete responses during weighting)
12(d)	If applicable, describe analytical methods taking account of the sampling strategy	Weighted prevalence estimates and 95% CIs accounted for the two-stage cluster sampling design, as per GYTS-specified weighting procedures
12(e)	Describe any sensitivity analyses	Sensitivity analyses compared linear, exponential decay, polynomial, and joinpoint-based prediction models for smoking prevalence, assessing model fit (R²) and projections to 2025 and 2030
Results		
13(a)	Report numbers of individuals at each stage of study, e.g., numbers potentially eligible, examined for eligibility, confirmed eligible, included in the study, completing follow-up, and analyzed	Sample sizes were reported: 1,830 (2001), 3,829 (2007), 3,348 (2010), and 4,526 (2022). All eligible students in selected classes participated, with response rates of 82.1%-89.3%. No follow-up, as cross-sectional
13(b)	Give reasons for nonparticipation at each stage	Nonparticipation is reflected in response rates (82.1%-89.3%), but specific reasons (e.g., absenteeism, refusal) are not detailed, consistent with GYTS reporting
13(c)	Consider the use of a flow diagram	Not included, as the cross-sectional design and standardized GYTS methodology do not necessitate a flow diagram
14(a)	Give characteristics of study participants (e.g., demographic, clinical, social) and information on exposures and potential confounders	Participants were students aged 13-15 years. Gender-specific data are provided. Exposure to tobacco control policies (e.g., MPOWER) is discussed, but no clinical or social confounders are detailed
14(b)	Indicate the number of participants with missing data for each variable of interest	Not explicitly reported, suggesting GYTS weighting procedures handled missing data
15	Report outcomes, exposures, predictors, potential confounders, and effect modifiers. Give unadjusted estimates and, if applicable, confounder-adjusted estimates and their precision (e.g., 95% confidence interval). Make clear which confounders were adjusted for and why they were included	Outcomes (e.g., current smoking: 4.7% in 2001 to 2.9% in 2022) are reported with 95% CIs. Gender and policy milestones are predictors/effect modifiers. No confounder adjustments, as the study is ecological (Tables 1-5)
16(a)	Report other analyses done, e.g., analyses of subgroups and interactions, and sensitivity analyses	Gender-specific trends, joinpoint regression (identifying 2010 inflection), segmented regression (policy impacts), and sensitivity analyses of prediction models (linear vs. nonlinear) are reported (Tables 3-6)
Discussion		
17	Summarize key results with reference to study objectives	The discussion summarizes the decline in smoking prevalence (38.3% reduction), gender gap narrowing, and declining cessation indicators, linking these to policy impacts and dental public health implications, aligning with study objectives
18	Discuss limitations of the study, taking into account sources of potential bias or imprecision. Discuss both the direction and magnitude of any potential bias	Limitations include self-report bias, irregular survey intervals, ecological design, limited age range, and lack of emerging tobacco product data. Potential underreporting (especially among girls) and unmeasured confounders are noted
19	Give a cautious interpretation of results, considering objectives, limitations, multiplicity of analyses, results from similar studies, and other relevant evidence	The discussion cautiously interprets trends as policy-driven, compares findings to global studies, and acknowledges limitations (e.g., self-report bias). It emphasizes sustained policy needs and dental interventions
20	Discuss the generalizability (external validity) of the study results	Generalizability is limited to youth aged 13-15 years in Saudi Arabia. The study notes that findings may not apply to older adolescents or other regions due to cultural and policy differences
Other information		
21	Give the source of funding and the role of funders for the present study and, if applicable, for the original study on which the present article is based	No funding was received for this study, and no funders influenced the research
22	Give information on conflicts of interest	No conflicts of interest are declared

## References

[1] Nazir MA, Al-Ansari A, Abbasi N, Almas K (2019). Global prevalence of tobacco use in adolescents and its adverse oral health consequences. Open Access Maced J Med Sci.

[2] Hu T, Gall SL, Widome R (2020). Childhood/adolescent smoking and adult smoking and cessation: the International Childhood Cardiovascular Cohort (i3C) consortium. J Am Heart Assoc.

[3] Itumalla R, Aldhmadi B (2020). Combating tobacco use in Saudi Arabia: a review of recent initiatives. East Mediterr Health J.

[4] Monshi SS, Alanazi AM, Alzahrani AM (2023). Awareness and utilization of smoking cessation clinics in Saudi Arabia, findings from the 2019 Global Adult Tobacco Survey. Subst Abuse Treat Prev Policy.

[5] Ramadan M, Alhusseini N, Samhan L, Samhan S, Abbad T (2023). Tobacco control policies implementation and future lung cancer incidence in Saudi Arabia. A population-based study. Prev Med Rep.

[6] Gajendra S, McIntosh S, Ghosh S (2023). Effects of tobacco product use on oral health and the role of oral healthcare providers in cessation: a narrative review. Tob Induc Dis.

[7] Al-Bedah AM, Qureshi NA, Al-Guhaimani HI, Basahi JA (2010). The Global Youth Tobacco Survey - 2007. Comparison with the Global Youth Tobacco Survey 2001-2002 in Saudi Arabia. Saudi Med J.

[8] Alasqah I, Mahmud I, East L, Usher K (2019). A systematic review of the prevalence and risk factors of smoking among Saudi adolescents. Saudi Med J.

[9] (2025). World Health Organization. Global Youth Tobacco Survey (GYTS) data repository. https://extranet.who.int/ncdsmicrodata/index.php/catalog/GYTS/.

[10] (2025). World Health Organization. Global Youth Tobacco Survey (GYTS). https://www.who.int/teams/noncommunicable-diseases/surveillance/systems-tools/global-youth-tobacco-survey.

[11] Hawkins SS, Bach N, Baum CF (2016). Impact of tobacco control policies on adolescent smoking. J Adolesc Health.

[12] Hawkins SS, Bach N, Baum CF (2018). Impact of tobacco control policies on adolescent smokeless tobacco and cigar use: a difference-in-differences approach. BMC Public Health.

[13] Levin SR, Hawkins SS, Farber A (2021). Association of state tobacco control policies with active smoking at the time of intervention for intermittent claudication. J Vasc Surg.

[14] Ngo A, Cheng KW, Chaloupka FJ, Shang C (2017). The effect of MPOWER scores on cigarette smoking prevalence and consumption. Prev Med.

[15] Taheri N, Fattahi P, Saeedi E (2024). A decade of tobacco control efforts: implications for tobacco smoking prevalence in Eastern Mediterranean countries. PLoS One.

[16] Amaral AL, da Costa Andrade PA, Lwaleed BA, Andrade SA (2023). Impacts of smoking on oral health-what is the role of the dental team in smoking cessation?. Evid Based Dent.

[17] Husain MJ, Datta BK, Nargis N (2021). Revisiting the association between worldwide implementation of the MPOWER package and smoking prevalence, 2008-2017. Tob Control.

[18] Lewit EM, Hyland A, Kerrebrock N, Cummings KM (1997). Price, public policy, and smoking in young people. Tob Control.

[19] Grucza RA, Plunk AD, Hipp PR, Cavazos-Rehg P, Krauss MJ, Brownson RC, Bierut LJ (2013). Long-term effects of laws governing youth access to tobacco. Am J Public Health.

[20] DiFranza JR, Savageau JA, Fletcher KE (2009). Enforcement of underage sales laws as a predictor of daily smoking among adolescents: a national study. BMC Public Health.

[21] Elharrar X, Fortin M, Beguinot E, Dols AM, Greillier L, Martinet Y (2019). Prohibition of tobacco sales to minors in France and Quebec. Comparison of legislative frameworks, their enforcement, and underage smoking. [Article in French]. Rev Epidemiol Sante Publique.

[22] Halboub E, Jafer MA, Khormi HI (2022). Attitudes and practices of tobacco cessation counseling among Saudi dental professionals: a nationwide cross-sectional survey. Niger J Clin Pract.

[23] Chan HL, Chan AK, Chu CH, Tsang YC (2023). Smoking cessation in dental setting: a narrative review on dental professionals' attitude, preparedness, practices and barriers. Front Oral Health.

[24] Harris M, Martin M, Yazidjoglou A, Ford L, Lucas RM, Newman E, Banks E (2022). Smokers increasingly motivated and able to quit as smoking prevalence falls: umbrella and systematic review of evidence relevant to the "hardening hypothesis," considering transcendence of manufactured doubt. Nicotine Tob Res.

[25] Skinner A, Occhipinti JA, Osgood ND (2021). A dynamic modelling analysis of the impact of tobacco control programs on population-level nicotine dependence. Sci Rep.

[26] (2025). Vision 2030. Health sector transformation program. https://www.vision2030.gov.sa/en/explore/programs/health-sector-transformation-program.

